# Shaping the Qualities, Values and Standards of Science. How Reporting Guidelines Improve the Transparency of Biomedical Research

**DOI:** 10.3389/frma.2022.846822

**Published:** 2022-06-27

**Authors:** Alexander Schniedermann

**Affiliations:** ^1^German Centre for Higher Education Research and Science Studies (DZHW), Berlin, Germany; ^2^Centre for Science and Technology Studies (CWTS), Leiden, Netherlands

**Keywords:** PRISMA, standardization, systematic reviews, scientific quality, transparency, reporting, science evaluation, reporting guideline

## Abstract

What is scientific quality and how can it be achieved? Recent developments in clinical biomedicine gave prominence to transparency as a new core value for scientific research. Without transparency, other characteristics and values remain unknown. But how can abstract concepts and values be implemented in day-to-day scientific practices and what gets lost on the way? In order to answer this question, this study investigates the role of the PRISMA reporting guideline for writing systematic reviews and meta-analyses. By combining a document analysis and expert interviews with its developers, it attempts to bridge the gap between research practice and current modes of evaluation. Beside showing how the guideline was designed to be applicable and acceptable as a new standard, the analysis revealed crucial distinctions between transparency as an abstract quality goal and its practical implementation in the form of specifically formulated rules. Although PRISMA relies on transparency in order to be meaningful, it blurs the concept in order to circumvent some of its main disadvantages.

## Introduction

The question of what research quality is and how it can be achieved is currently one of the most debated issues among scientists, funders and policymakers. Recent developments highlighted concepts such as research integrity or responsible research that give prominence to more alternative notions of research quality (Langfeldt et al., 2019). One of such alternative quality goals that has gained increasing attention is transparency. Originating from philosophical discussions about the proper epistemic values for science, scholars soonly placed transparency among other quality criteria such as plausibility, reliability or credibility and created what sometimes has been called “a tapestry of values” (Elliott, 2017) or value “portfolio” (Mårtensson et al., 2016). Such a tapestry not only visualizes the complexity of the aims and goals in science, but also how there can be multiple and even conflicting notions of research quality at the same time (Petersohn et al., 2020).

In practice, transparency is related to a multitude of initiatives and developments in various scientific disciplines. Open access publishing removes paywalls and, in principle, makes research results available to wider publics (Nosek and Bar-Anan, 2012; John, 2017). Open data or open code promise to lift the curtains of individual research projects in a similar manner (Leonelli and Tempini, 2020). In addition, scientists, funders and policymakers attempt to change the evaluative culture of science by demanding a more pluralistic and transparently communicated set of quality goals for research (Langfeldt et al., 2019). As such, peer review becomes more transparent because journals publish review reports and editorial decisions (Hartstein and Blümel, 2021; Waltman et al., 2022). In general, standardized forms of research assessment, e.g., ex ante assessments of grants and job applications (Hammarfelt and Rushforth, 2017), or ex post evaluations in peer review or bibliometric assessment, promise transparency by making evaluative procedures traceable and comprehensible (Petersohn et al., 2020). Combined under the label of “Open Science,” scholars hope that making various aspects of the research process more transparent increases public trust and accountability, leading to a “credibility revolution” (Vazire, 2018, p. 411; see also Fecher and Friesike, 2014).

In clinical biomedicine, transparency would not only increase the credibility of research, but also improve medical treatment to the benefit of patients. While often overlooked in the history of Open Science, method experts became engaged with these issues even before the 1990's. Analogous to treatment guidelines, they developed so-called “reporting guidelines” that instruct researchers in writing their articles, so that these are appraisable and relevant to medical practice and can serve as evidence for evidence-based medicine (Rennie, 1995; Green et al., 2009; Ioannidis, 2016). Beside the famous CONSORT guideline for randomized-controlled trials, especially the “preferred reporting items for systematic reviews and meta-analyses,” or shorter, “the PRISMA Statement” became well established (Moher et al., 2009). Like other reporting guidelines, PRISMA consists mainly of a checklist with 26 items or reporting rules that tell authors what information about the performed study are to be included in the final publication.

As the name suggests, PRISMA aims specifically at systematic reviews, a highly standardized version of research synthesis that promises to rule out various biases by employing specific techniques for searching and appraising primary research (Moreira, 2007). One major task in the conduct of such studies is the systematic search and filtering of primary research. To become 'systematic', this task has to be based on pre-defined search terms and inclusion criteria so that it becomes reproducible (Hunt, 1999). The PRISMA guideline not only consists of the reporting checklist, but also of a flowchart that visualizes how the number of included studies decreases with every applied criteria.

Due to their high level of formalization and technical standardization, systematic reviews and meta-analysis are supposed to prevent subjective study selections and rule out various other biases as well (Chalmers et al., 2002). For this reason, this genre became increasingly popular during the recent history of medicine and gained a lot of attention due to the formation of the Cochrane Collaboration which mainly focuses on the production of systematic reviews. Especially during the 1990's, systematic reviews were knighted as the representatives of the primary source of evidence in evidence-based medicine and were placed on top of the hierarchy of evidence (Timmermans and Angell, 2001; Stegenga, 2011).

PRISMA and other reporting guidelines transform the rather abstract quality goal of transparency into a very distinct standard that attempts to change and guide the behavior of authors of systematic reviews. Traditionally, standards are understood as tacit or codified forms of shared practices that demarcate science from other societal spheres (Kuhn, 1962; Crane, 1972; Latour, 1987 [1962]; Whitley, 2000 [1987]). In clinical medicine, the standardization of research questions, vocabulary, instruments or methods was a crucial factor in defining the discipline and equipping it with its unique translational character (Roth, forthcoming; Fujimura, 1987; Timmermans and Berg, 2003). Beside their community-forming function, standards provide interpretations of existing or new quality goals for a particular domain and are often encountered in attempts to implement any form of quality assurance or quality management (de Jonge et al., 2011). But in defining quality goals, standards also perform boundary work and thus can spark substantial frictions with existing practices, especially if a new standard claims wide application (Hallström, 2002). Therefore, reporting guidelines and other Open Science initiatives differ from many other standardizations in science due to their attempt to introduce new notions of research quality and reshuffle the tapestry of values.

While there are many studies about how standards reshape scientific practices and enable community building, distant communication or education, less is known about how standards interact with scientific quality goals and epistemic values. Usually, scholars argue how science is defined by a very particular set of epistemic values that make it so unmistakable successful in its collective strive for knowledge (Goldenberg, 2009; Elliott and Steel, 2017). In addition, the “value-free ideal” suggests that political, industrial, or other “social” values do not influence science (Steel, 2010). But as mentioned earlier, there is a palette of values and quality goals at once and studies have shown how these shape research practices (Müller and de Rijcke, 2017; Aksnes et al., 2019). Accordingly, scholars argued how changing values and quality goals means to set new incentives or rules while ending existing ones (Nosek et al., 2013).

In order to understand how a new standard affects the quality goals of research, this study investigates the characteristics of the PRISMA guideline and its development. It aims at bridging the gap between the theoretical discussions of transparency and actual research practices. In asking how transparency becomes manifest in the formulations and rules included in the guideline, a practical case of how quality goals and research values are introduced in science is provided. In other words, this study attempts to illuminate the relation between doing science and communicating scientific results and how public expectations or quality goals can influence the latter. Especially since there are already quality goals for doing science, this study goes beyond the analysis of the mere emergence of transparency as a new value and investigates how transparency is aligned with the existing quality goals and evaluative cultures.

Together with these topics, this analysis attempts to shed light on how new values and practices affect the current modes of research assessment. Since PRISMA impacts the written outputs of science, it presumably not only interferes with dominant practices and cultures in research, but also evaluation procedures such as peer review. Therefore, an analysis of PRISMA supports our understanding of the link between standards for research, their inscribed epistemic values, and cultures of research evaluation. While the last two are often discussed with regards to innovations in Open Science, especially the standard as practical implementation of both can shed light on what research qualities become assessable or meaningful at all.

## Data and Methods

To understand the characteristics of PRISMA, a qualitative approach that combines a document analysis with qualitative interviews was chosen. This double approach allows to make claims about the guideline as a document, while, at the same time, reduce the impact of some biases of document analysis, such as insufficient detail and selectivity (Bowen, 2009).

In a first step, all versions and publications of PRISMA documents, their explanatory supplements, as well as translations were retrieved from the guideline's website (www.prisma-statement.org), the EQUATOR network (www.equator-network.org), as well as a Web of Science version provided by the German Kompetenzzentrum Bibliometrie (www.bibliometrie.info). Specific characteristics of the PRISMA documents were then highlighted during an open coding process. In this process, not only contents and narratives were identified. Rather, this process also captured meta data that is meaningful in relation to scientific publishing, such as authors, affiliations, publishing journals, citation impact. In addition, also the length and extensiveness of the guideline documents was assessed. Since meta data of scientific publications usually represents the high level of standardization in formal scientific communication, deviations become meaningful as well. For example, the multi-publication of the guideline documents, or the distinction between authors and mere workgroup members were analyzed and interpreted.

Based on the list of authors and workgroup members, potential candidates for expert interviews were identified and contacted. Issues such as personal or professional diversity of interview candidates were considered during the selection process. Interview guidelines were informed by theoretical insights about standardization processes, e.g., the role of different actors and contexts or questions of authority and power. Yet more importantly, the guideline was informed by the results from the document analysis so that the interviews could be used to inform the interpretation of PRISMA's special characteristics.

Two interviewers performed seven semi-structured interviews with its developers during the first quarter of 2021. Candidates were chosen from authors, workgroup members and translators of PRISMA or one of its updates. During the interviews, participants were asked about their professional background and their role in the development process of the guideline. Interviews were recorded and transcribed by a third-party service that combines automatic and manual methods for transcription. Written transcripts were anonymized and then analyzed by using MAXQDA and Microsoft Excel. In case of ambiguities, transcripts and audio recordings were cross-checked. Audio recordings will be deleted at the beginning of May 2025.

Two interview participants were involved as authors on PRISMA's earliest version published in 1999 (Participants B and C), three authored its 2020 version (Participants E, F and G), one authored the main version from 2009 (Participant A) and one authored its German translation (Participant D). However, most interviewees also contributed to the other versions as workgroup members without being listed as author afterwards. All participants were prolific and highly-cited researchers in clinical research where they focused on topics such as research ethics, research design, statistics or information retrieval. Notably, one participant has built an extensive profile as editor of some of the most prolific biomedical journals (Participant B), one has also substantial experience in industry (Participant C), and three are also associated with the Cochrane Collaboration (Participants A, B, F).

The coding of the interview transcripts was guided by several deductive codes that were developed from the results of the document analysis, previous research about scientific communities, the standardization of epistemic practices in general, as well as the development of guidelines and checklists in particular. However, results from the analysis of deductive codes have been reported at length elsewhere and will only occasionally be mentioned here (Schniedermann et al., 2022). In addition, the qualitative analysis also employed exploratory open coding procedures in order to identify additional topics and particularities. One of those topics was the role of transparency for the guideline. Since this may only serve as a legitimating narrative, the coding tried to consider any conceptual plurality or disagreement between participants. The results of this analysis are provided in this study.

## Results: Standardizing Science

Since the inception of its first version in 1999, PRISMA has become one of the most widely disseminated and adopted reporting guidelines. It accumulated over 35k citations which makes it not only the most cited reporting guideline (Caulley et al., 2020). Rather, its over 11k citations from systematic reviews show its wide dissemination and acceptance across various disciplines in clinical medicine (Schniedermann, 2021). To utilize this significance for the better, metascientists analyze the level of compliance and identify shortcomings and usage barriers (e.g., Moher et al., 2010; Page and Moher, 2017; Peterson and Panofsky, 2020). Subsequently, its developers update the guideline and provide new versions, the latest being from 2020 (Page et al., 2021). Besides its high citation impact, PRISMA is endorsed by several journals and is in any way implemented into their editorial practices which will be elaborated later.

On a first glimpse, the PRISMA guideline are short documents that resemble the typical form of journal publications. A second look reveals how they are embedded in a wider ecosystem of guideline documents and their updates, experts and journals. While its first version was published once in *The Lancet* in 1999, its most cited version was published in seven different journals in 2009 while its last release was published in five different journals and as a preprint in 2020. Since 2009, these fourteen rather similar versions are accompanied by overall six publications with additional explanatory material and examples (e.g., Liberati et al., 2009)^1^. In addition, the PRISMA statement was officially translated into German, Italian, Portuguese, Spanish, Japanese, Italian and Croatian, while its checklist and flowchart were translated into other languages as well. During its course of development, its size grew from six broader topics in 1999 to twenty-seven distinct and specific rules in 2009 and some additional sub-rules and checklists in 2020.

In its introduction, the PRISMA guideline problematizes the current reporting of systematic reviews as improper and offers a set of rules as a viable solution. But instead of picturing ideal forms of scientific writing against which existing publications can be evaluated in a binary matter, the guideline rather accounts for the variance in medical reporting and how it makes some but not all systematic reviews useless for medical decision making. Based on studies about this variance, it notes that “the reporting quality of systematic reviews varies, limiting readers' ability to assess the strengths and weaknesses of those reviews” (Moher et al., 2009) which complies with the main rationale for reporting guidelines as usually discussed (Rennie, 1995; Altman and Moher, 2014; Ioannidis, 2016). Likewise, interviewees have noted how the guideline is kept to the bare minimum in order to set the lower end of necessary reporting:

“I feel like people should not always be told what to do, but be creative of doing whatever they're doing. Unfortunately, you're assuming everybody is at certain level. You are assuming people would know how to write a paper, but that's not the case. […] I think that's a very good example, why standards are needed for many of the things we do. It's about reproducibility. It's about using the same terminology and language to communicate with each other. It's about transparency in science and I would say most of these standards are sort of minimum standards” (Participant F)

Another interview participant explained how the definition of the bare minimum was inspired by the standardization of medical practice that can be found in the form of checklists that are developed to prevent the most harmful events, rather than ensuring good practice in general:

“So, mistakes can be made, ludicrous mistakes, operate, took off the wrong leg. Well, that's a big joke to everybody except the patient and in fact the surgeon. And so, a simple checklist makes that nearly impossible.... [and] I then saw that exactly the same principles might occur if you made writing a paper... following the requirements, that you state exactly where you give the following bits of information.” (Participant B)

The attempt to define the minimum rather than the optimum not only increased the potential acceptance of the guideline, but also its implementation into the formal submission requirements of scientific journals. Similar to the introduction of the IMRAD structure of scientific articles (Rennie, 1995), or the more recent effort to widely establish the pre-registration of trials (Altman and Moher, 2014), guideline developers faced the transparency crisis in medical research by asking the question “what can journals do?” (Altman, 2002, p. 2765), in order to improve the written outputs of scientific practices. Thus, the developers actively enrolled scientific journals in the development and dissemination of PRISMA and thereby went beyond the mere promise of methodological advancement. During the interviews, two benefits of the participation of journal editors in the development of the guideline became apparent.

First, writing and publishing is an activity that lies in the domain of academic journals and the expertise of journal editors. They can inform the guideline developers about formal requirements of academic publishing and its trajectories. For example, one interviewee mentioned how the limited space of print journals and the editors' calls to reduce supplementary material initially worsened the situation of reporting since there was simply no space for extensive information. In addition, involving journal editors provided the required representation of this group in order to make them accepting the guideline. In that sense, journals take substantial responsibility for the state of written science.

Second, by enrolling the “gatekeepers of science” (Crane, 1967, p. 195) and make them endorse PRISMA equips it with a level of regulatory authority that goes beyond the mere argument or evidence for the superiority of one method over another. Instead, the workgroup intended to implement the PRISMA guideline more directly into the formal requirements of academic publishing such as submission guidelines, editorial decisionmaking and the peer review system. As a result, the successful application of the guideline would not alone rest on the author's belief that compliance is useful. Instead, turning compliance with PRISMA into a submission requirement can be seen as some level of regulatory enforcement (Schniedermann et al., 2022). However, interview participants have mentioned that, originally, the workgroup hoped most journals would require the filled-out checklists from authors. But there is substantial variance in how journals deal with these, as the following quote suggests:

“[R]esearchers will check off and indicate where in a paper they have complied with a specific reporting item. And then that checklist can get attached to the article. So that was an original idea and some journals do that. And often, some journals will collect that, but they don't actually publish it. (Participant B)”

In making guideline compliance a formal requirement of publishing systematic reviews, the guideline becomes part of the evaluative regimes in research assessment. The judgements about whether a systematic review is accepted for publication or not can be seen as a form of *ex post* evaluation, or quality control, that filters out any submissions considered of too low quality. This usually happens during peer review in which the evaluators' motivations and criteria are mostly rooted in tacit knowledge and remain unknown to outsiders. Since these criteria can be subjective or even biased, formal requirements such as guideline compliance can be employed even before peer review starts (Hojat et al., 2003). In both cases however, the evaluation is *ex post*, because it is performed after the research project was finished and the final report has been written.

In implementing PRISMA into the spheres of research evaluation, the guideline transforms the genre in the long run and thereby evaluates research *ex ante* as well. If compliance with PRISMA becomes a necessary condition to pass or even enter peer review, in some journals, non-complying systematic reviews will slowly disappear. This is because authors will comply with the guideline out of convenience, once they learned its requirements and techniques. Now, even when a particular journal does not formally require guideline compliance, the mere expectation of the authors that journals in general require compliance leads to a proliferation of the new standard. Therefore, similar to how researchers incorporate the dimensions of research evaluation into their epistemic practices (e.g., de Rijcke et al., 2016), PRISMA compliance becomes a meaningful milestone during the writing of systematic reviews. As it will be explained below, its requirements may even influence the actual conduct of studies even before writing starts. However, in becoming an *ex ante* evaluation as well, PRISMA not only enables the discrimination between transparent and non-transparent reports, but initiates a complete redefinition of the systematic review genre. During the interviews, it became clear that especially high impact journals spearhead this redefinition by appointing specific editors for guideline compliance and similar tasks. In addition, the potential to automatically check guideline compliance with the help of editorial software systems will accelerate this development.

New standards in scientific communication perpetually redefine what counts as legitimate knowledge and PRISMA can be understood this way as well. Scholars who investigated standardization have argued that it not only forms communities by representing consensus on a shared set of practices, but also enables the distant communication between different actors and contexts (Fujimura, 1988; Bowker and Star, 1999; Timmermans and Epstein, 2010). This is especially important to scientific communication which requires the de-contextualization of scientific practices by offering “a way to harness stories of the smaller world of the laboratory to general claims about the regularities of the larger world of nature” (Bazerman, 1988, p. 79). In that sense, especially clinical medicine developed a range of standardized genres such as the randomized controlled trial which became the “gold standard” (Timmermans and Berg, 2003) and meta-analyses which is sometimes labeled as the “platinum standard” (Stegenga, 2011) in modern biomedicine and is equipped with substantial political and epistemic authority (Swales, 2004; Csiszar, 2020). Yet, in contrast to these preceding standards, PRISMA is offered in a much more straightforward and codified form and suggests a strong influence by guidelines for medical practices, but resembles usual methodological advancement through new textbooks and formal training only to a lesser extent (Schniedermann et al., 2022).

## Building Transparency

PRISMA can be understood as a practical manifestation of transparency in scientific reporting. Although a quality goal such as transparency may be in fact abstract and open to very different interpretations (see Langfeldt et al., 2019), its translation into a practical checklist with rules to follow inhibits a particular definition of what transparency is and how it can be achieved. As already mentioned, it consists of twenty-seven different rules that start from choosing insightful titles and provide clear discussion and limitations. But most of its rules focus on very specific aspects of reporting medical research in general and systematic reviewing in particular. For example, item 12 requires authors to

“Describe methods used for assessing risk of bias of individual studies (including specification of whether this was done at the study or outcome level), and how this information is to be used in any data synthesis” (Moher et al., 2009, p. 5)

By aiming at very specific steps in the making of a systematic review where authors have to make several decisions, the PRISMA reporting guideline establishes a connection between the final report and the process of conducting a systematic review that usually happens in what has been called a “textual laboratory” (Moreira, 2007, p. 185). But for authors, the “laboratory” rules and checklists do not only provide a clear and straightforward advice about the next steps and tasks that have to be done (see also Stegenga, 2011). Rather they become an efficient device for effectively achieving the quality goals that are required by the biomedical community such as increased transparency (Fujimura, 1988). In the end, not only individual authors but also the community as a whole benefits from such a codified standard.

By subordinating the guideline under transparency as an abstract quality goal and employing the wider narrative about the transparency crisis, PRISMA helps to foster and further perpetuate the autonomy and public legitimization of biomedical research. For example, treatment guidelines promise to ensure proper cost control of medical practice. But if doctors do not comply sufficiently, the wider public can accuse medicine of being wasteful, especially if it is funded with tax money (Timmermans and Berg, 2003). Similarly, standards like PRISMA equip biomedical research with a form of professional jurisdiction that becomes visible from the outside and is also comprehensible due to its abstract narrative about transparency. It serves as a proof that researchers are not only aware of the current problems and crises in their domain, but also attempt to solve these issues so that there is no need for any form of intervention by the wider public. In that sense, interviewees often mentioned how it is an ethical duty to apply PRISMA:

“So, I would rather say that it is unethical to do research the way it has been done for a long time. Where one can then say that certain studies simply do not meet the standards, do not meet a sufficient quality. And that means we have a problem for society. We have a problem with the patients who are in the studies. We have a problem with the animals that were used in experiments and whose data were worthless in the end. So, we have a very big responsibility” (Participant D, machine translation)

PRISMA and other reporting guidelines became visible to outsiders by incorporating their core values into the common intersections between clinical research and publics such policymaking, medical doctors, patients or other academic fields. Considering it as an aspect of research ethics, reporting guidelines re-defined what should be evaluated as good research. New cultures of research evaluation have become especially visible in the form of manifestos, such as the Leiden Manifesto or the San Francisco Declaration on Research Assessment (Leckert, 2021). Not surprisingly, medical experts extended these efforts by formulating the “Hong Kong Principles for assessing researchers” (Moher et al., 2020). This manifesto stresses the importance of transparent reporting and subsequently turns reporting guidelines into its second principle. It addresses especially funders but also individual institutions when asking for a better implementation of such guidelines.

PRISMA and its incorporation into a framework such as the Hong Kong Principles promises a more accountable form of research evaluation. Scholars have mentioned how research policymakers and funding bodies become more inclined toward standardized forms of research evaluation and actively support the development of various frameworks (Mejlgaard et al., 2020; Petersohn et al., 2020). Especially the standards' claim to properly capture aspects such as credibility, rigor or transparency serves as their promise to incentivize the right trajectories for science, rather than just any (Langfeldt et al., 2019; Peterson and Panofsky, 2020). Instead of the traditional focus on outcomes such as publication or citation numbers in order to define excellent research, standards can provide a form of regulation in which the wanted goals and qualities are incorporated into epistemic practices (Freese and Peterson, 2018). In turn, effortful quality evaluations can be substituted by evaluations of guideline compliance. Therefore, PRISMA makes not only reporting more transparent, but also research evaluation if it is incorporated in evaluative frameworks.

Using standards and standard compliance as proxies for any form of evaluation increases bureaucratic burden even if the standard is limited to a minimum, as PRISMA's developer have argued. On the level of the individual systematic review that has to pass through editorial offices and peer review, compliance with PRISMA results in filled-out checklists and flowcharts that have to be reviewed. During the interviews, the developers of PRISMA mentioned how compliance checks consume additional time of reviewers and thereby worsen the situation of the already overburdened peer review system in biomedicine (see also Kovanis et al., 2016). In addition, they mentioned how peer review was not able to prevent the problems which PRISMA aims to solve in the first place. Thus, the additional burden of reviewing guideline compliance should best be implemented at editorial offices:

“Again, as I told you, it's a minimal guideline and then journals have to allocate resources to check the compliance. You cannot really rely on the unpaid peer reviewers to do any of this. You have to have dedicated in-house staff whose job is to check the compliance to the guidelines. Unless this becomes a paid job of someone, it's not going to happen.” (Participant F)

“I send my report to the funder as good practice. Does someone in the funder's office then sit down and check, go against the checklist and see if I have done so? I don't know. I think some funders might, but I think many funders would just not want that bureaucracy” (Participant A)

This suggests that the success of the standard can become its own undoing because the sheer amount of available information may make a systematic review project not more but less transparent. This becomes especially important if we consider why transparency was promoted in the first place. Instead of trying to answer how good research looks like, e.g., how to properly conduct a systematic review, experts shifted their efforts towards a standard for reporting and writing of reviews. Seemingly, transparency promises that there is no need to decide proper conduct now or ever. Instead, knowledge users and readers are served with more information about the processes behind the review and have to judge the quality of conduct in each individual case, which is often called the “backtracking” function of transparency (McKaughan and Elliott, 2013). But in actually performing backtracking, knowledge users have to reassess the whole process from the data to the conclusions and judge the authors' decisions against their own set of quality criteria which is a laborious task (Elliott, 2020). In contrast, standardization usually combines and packages methods and tools so that these can be used efficiently by other scholars who do not have to validate the very foundations of the standard by their own means (Fujimura, 1987, 1992). This perspective on standardization stresses how standards usually reduces individual efforts and thereby contradicts the ideas of transparency and backtracking to some extent.

## How Prisma Blurs the Concept of Transparency

The most crucial feature of PRISMA and similar guidelines is the often-stressed distinction between reporting and conduct. While the latter captures the process of doing a study such as a systematic review, the former only addresses the writing or reporting of results. Although this distinction proves to be rather fuzzy in practice which will be discussed below, it is crucial for the acceptance and applicability of the guideline. Since its 2009 version, this distinction is elaborated in an additional info box (Moher et al., 2009, p. 2). However, the biomedical research community seemingly too often confused this distinction so that experts argued how: “a further confusion between reporting and conduct emanates from the misuse of reporting guidelines. This misuse often takes the form of researchers using a guideline to develop a quality score for conduct of studies” (Schulz et al., 2014). Subsequently, the 2020 version of the guideline further accentuates the distinction by stating:

“PRISMA 2020 is not intended to guide systematic review conduct, for which comprehensive resources are available. […] However, familiarity with PRISMA 2020 is useful when planning and conducting systematic reviews to ensure that all recommended information is captured. PRISMA 2020 should not be used to assess the conduct or methodological quality of systematic reviews; other tools exist for this purpose.” (Page et al., 2021, p. 2).

The main idea behind separating reporting from conduct is that both categories can be evaluated individually. Experts argue that first and foremost, the various methodological decisions during systematic reviewing, for instance study design, inclusion and exclusion criteria or statistical techniques have to be reported thoroughly, regardless of which decisions actually have been made (Chalmers and Glasziou, 2009; Altman and Moher, 2014). In that sense, the guideline does not inform any evaluation about whether a study was appropriately conducted or not. Even more complex, its underlying rationale suggests that the judgement of whether a study was conducted appropriately is highly relative, thus not independently decidable. Clinical disciplines may vary a lot in their individual expectations about methodological soundness.

Developing a guideline that did not interfere with the variety of conceptions about proper conduct would reduce the potential of coming at conflicts with other, local or disciplinary standards. It makes PRISMA not only applicable because it aligns with predominant research cultures but also acceptable because it minimizes the requirement to change individual beliefs about what appropriate research practices are (Schniedermann et al., 2022; see also Timmermans and Epstein, 2010). For example, the prominence that systematic reviews gained due to the shift toward more evidence-based medicine sparked substantial controversy in scientific fields that employ qualitative research, such as nursing (Porter and O'Halloran, 2009; Jovanović, 2011). Even highly quantitatively disciplines initially struggled with addressing heterogeneity, as the following quote suggests:

“But in observational, in case control and in cohort studies, everybody makes up their own methodology. I mean, in case control studies, for example, you might have one study on treatment of breast cancer that matches participants on a three to one basis, and then you'll have another study that matches on like two to one basis. Well, how do you pool those results?” (Participant C)

Likewise, medical doctors are not only interested in the methodological rigor of a study, but whether it is relevant to their individual patient and thereby sparked debate over external validity of systematic reviews. If they treat an aged patient, doctors find it more appropriate to consider evidence from studies were elderly participants took part (Avellar et al., 2017; see also Cartwright, 2007). Therefore, the individual appraisal of a systematic review is highly context dependent and can vary a lot by user group (Moreira, 2005; Liberati et al., 2009). Even within the same group, conceptions of appropriate conduct may change over time but transparent reports allow for a re-appraisal.

The boundary between reporting and conduct was also crucial for PRISMA's successful and cost-effective development. Limiting the efforts to a small part of the whole research process–the writing of a particular type of scientific publications–helped the developers to limit the required expertise and resource in order to develop a short, yet comprehensive reporting guideline. Interview participants referred to those “comprehensive resources” when they explained how there are many different and well-established standards and methods for conducting systematic reviews and that their group had no intent to extend this list. Especially in the case of systematic reviewing, the role of the Cochrane Collaboration becomes important in this regard. By providing extensive standards and rigorous guidance for conducting, reporting, editing and publishing, Cochrane covers the whole pipeline in doing systematic reviewing (see Chalmers, 1993). By targeting reporting only, the developers of PRISMA not only limited their efforts, but also set the intellectual and organizational boundaries against the Cochrane Collaboration.

The distinction between reporting and conduct not only limited the developers' efforts, but also enables a level of professional jurisdiction that is doable and acceptable. For PRISMA, this means that the strict limitation to reporting makes the standard enforceable by the editorial offices of academic journals. As already shown above, the implementation of PRSMA into editorial or peer review was an initial aspect of its design. Consequentially, the guideline developers involved journal editors and evaluated the boundaries of how far journals can go and what they can demand from authors without interfering too much with disciplinary idiosyncrasies and thereby limiting epistemic pluralism (Schniedermann et al., 2022).

At the same time, the limitation to reporting and the enforceability by journals initially transforms transparency from an abstract concept into an evaluative category. By interpreting the nature of transparency in the light of particular decisions, one can decide whether some actions become transparent or not (Elliott, 2020). This is what has been understood as the practical manifestation of an abstract quality goal in this essay. Especially in the case of transparency, such a manifestation becomes a valuable target of investigation because transparency is often conceptualized as a relative concept or meta-value that enables the manifestation of other values (Turilli and Floridi, 2009). Therefore, PRISMA equips the concept of transparency with a specific meaning in the first place and it becomes rather obvious why its developers stress the distinction between reporting and conduct.

In practice, the distinction between reporting and conduct is much fuzzier. The authors of the guideline have admitted fuzzy boundaries in the case of systematic reviews because these are essentially performed on the researchers' desks and do not involve laboratory or clinical practices (see above). But there are several other factors as well. Advocates of transparency in general and reporting guidelines in particular have elaborated their hope that more transparency or better reporting will lead to better conduct in the end (Schulz et al., 2014; Vazire, 2017). Comparing science to a market, Simine Vazire argues that “the fact the fact that buyers could potentially detect many misrepresentations would make ’sellers' (i.e., authors) much more accountable, and would likely increase the care with which authors conduct their studies and write up their results” (2017, p. 3). Thus, some functional or causal effects are even emphasized in the conceptual considerations of how biomedicine benefits from greater transparency.

In the case of PRISMA, interview participants have made similar assertions. Instead of discussing promises or intentions, they explained that the way how the guideline's rules are formulated bears the functional connection between reporting and conduct:

“But there's also a good, I guess, it's a fair assumption that [...] once you've thought about what needs to be reported and documented in a review, that you're going to be paying closer attention to those details when you're actually conducting a review” (Participant E)

“[T]hey're there to instruct authors about what they should report. But when […] you read between the lines, it's obvious, it's quite clear that there are certain expectations about how authors should approach and conduct their review just in the way in which the guidelines are written.” (Participant G)

This fuzziness between reporting and conduct in the PRISMA guideline shall be demonstrated with three more examples. First, PRISMA requires authors to make some statement about whether there is any pre-registered protocol in which the authors of the systematic review have prefigured the review and described their research questions and study design:

“Indicate if a review protocol exists, if and where it can be accessed (such as web address), and, if available, provide registration information including registration number” (Moher et al., 2009, Item No. 5).

One expert mentioned how there was no such registry for systematic reviews at the time when the guideline was published, so that the some of the developers went on to establish the PROSPERO registry (see also Page et al., 2018). He further noted that: “[...] PRISMA said you should give the registration number review, which then made people think, well, therefore we should register our review prospectively” (Participant A).

Second, the guideline requires authors to “Describe the methods of handling data and combining results of studies, if done, including measures of consistency (such as I^2^ statistic) for each meta-analysis” (Moher et al., 2009, Item No. 14). The way of how the rule is formulated not only that there are specific methods for combining data, but also very particular concepts such as the measure of consistency. Respectively, one interviewee explained that “[...] if somebody is doing a meta-analysis [...] they say, ’oh, I've got to assess heterogeneity because that's going to be required in my report” (Participant C).

Third, PRISMA demands authors to “specify any assessment of risk of bias that may affect the cumulative evidence (such as publication bias, selective reporting within studies)” and “present data on risk of bias of each study and, if available, any outcome-level assessment (see item 12)” (Moher et al., 2009, Item No. 15 and 19). In such assessments, researchers have to estimate the potential biases involved in the primary studies that will be included or excluded from the review, in order to make a judgement about the reliability of the outcome. This well-established practice often itself involves standardized assessment scales and tools (e.g., Whiting et al., 2016). However, one interview participant mentioned that

“There could be some elements that people might start interpreting, so you should describe what risk of bias or quality assessment tool you used, makes me think I should therefore be using one” (Participant A)

By setting up specific rules for reporting, the guidelines define what has to be transparently reported and thereby point toward certain expectations of how systematic reviews are properly conducted. Similar relations can be found elsewhere. For example, the requirement to report “conflicts of interests,” which became a usual characteristic of clinical research, does not explicitly devalue industry funded science, but stems from the discovery that such research is more likely to be biased (Als-Nielsen et al., 2003; Jørgensen et al., 2006; Michaels, 2008). Instead of generally prohibiting industry-funded research, it is assinged a “red-flag” that calls for a careful and accurate contextualization of results.

All in all, although the distinction between conduct and reporting is required to make transparency a meaningful quality goal that can be achieved by the regulatory capacities of academic journals, the actual rules and items of the guideline blur this distinction. In providing a list of study characteristics which have to be transparently reported, such guidelines inherently provide prominence to those characteristics. Put differently, in defining what has to be transparently reported, PRISMA promotes certain practices and decisions that lie beyond the writing of the report.

The violation of the distinction between conduct and reporting contradicts the concept of transparency, yet at the same time, also circumvents one of its major weaknesses. Discussing a more abstract interpretation of transparency, advocates hope that transparency as a quality goal helps to solve several issues of modern science, sometimes called “the new worries of science” (Kourany, 2020). With such worries, philosophers especially mean the meddling with science by something that is undeniably non-scientific. In more concrete terms, this refers to the growing influence of partisan politics or private industry on epistemic practices. Against the background assumption of a value-free ideal of science, governments are accused of avoiding inconvenient truths such as the climate crisis by defunding whole disciplines (Hoag, 2012), or pharmaceutical companies by burying study results that endanger their financial prospects (Michaels, 2008; Macleod et al., 2014). Notably, also the current mode of science policy is criticized for promoting inappropriate goals for science and incentivizing bad behaviors, so that experts demand a more responsible and meaningful research evaluation (Langfeldt et al., 2019).

But the interference of non-scientific interests with epistemic practices remains a complex problem that transparency aims to solve. Scholars have pointed out how even democratic majorities can consent on the wrong goals for science (Steel, 2017), or in contrast, political agendas can improve pluralism and innovativeness by empowering underrepresented groups (Kourany, 2020). From this background, it seems unlikely that the value-free ideal can be achieved. Instead, transparency promises to make all those potentially non-epistemic values and their influence on a research outcome visible. Analogously, transparent reporting makes the various steps of the research process visible and readers can backtrack the decisions that were made (see above). Readers or knowledge users then not only can estimate how a particular methodological decision may be skewed the study toward a particular conclusion. Rather, they become able to reprocess the research by incorporating other decisions or values and thereby receive a result that is normalized toward these decisions or values (Elliott, 2020).

Such a definition of transparency comes with new problems, as indicated above. The result of more transparent reporting imposes additional burden on peer reviewers, editorial offices and science funders. In general, it advocates for a more dynamic nature of scientific values and the requirement for ongoing negotiations, e.g., about proper conduct. It may help to circumvent debates over proper values in the first place, but only postpones such debates to a later stage. Similarly, if PRSIMA does not define expectations of proper conduct beforehand (which it does), those who consume the systematic review still have to answer this question in order to make a practical decision. Thus, the effort of evaluation would not have been reduced but just relocated.

The mere relocation of evaluative efforts shows how the very idea of avoiding conflicts over deciding proper conduct by focusing on transparency is fallible. Such an attempt misunderstands transparency itself as a sufficient quality goal for science, as portrayed in some value lists (e.g., Mårtensson et al., 2016). Instead, as mentioned before, transparency is more adequately interpreted as some form of meta-value or conditional quality (Turilli and Floridi, 2009). But being a meta-value, transparency is no meaningful evaluative category because it enables (and requires) the evaluation of other values. It is not helpful in the sense that it does not reduce evaluative efforts. The potential to evaluate science solely by reviewing transparency itself or monitoring guideline compliance is thus limited (see also John, 2017). Likewise, it has been argued that the sole focus on more transparency and a resulting increase of the disclosure of value judgements does not automatically ensure that the right judgements are made (Etzioni, 2010). Instead of focusing on transparency and its promises, philosophers have argued for a more modest approach, in which scientists should debate and consent on the values and quality goals that are politically or ethically acceptable (Kourany, 2010; Brown, 2018).

The PRISMA guideline does not serve such an abstract version of transparency. The guideline widely promotes transparency and stresses the distinction between conduct and reporting in order to be acceptable and applicable. At the same time, it incentivizes authors to make certain decisions during the conduct of their reviews, such as pre-registration, risk of bias assessment or estimate heterogeneity. To some extent, this was even intended by advocates and developers of reporting guidelines. This blurs the distinction between conduct and reporting and shows how transparency must be interpreted as meta-value that is not only required to define other quality goals, but itself depends on the existence of other qualities in order to be meaningful. Therefore, the role of transparency for the PRISMA guideline is twofold. On the hand, transparency is the nurturing narrative behind the guideline that is, on the other hand, purposefully violated in order to make the guideline explicit and useful in scientific practice.

If transparency allows for the application of PRISMA in various clinical subdisciplines while, at the same time, violates the distinction between conduct and reporting, it not only inhibits some conceptual tension itself, but may also sparks frictions with disciplinary idiosyncrasies and local cultures of research which it initially attempted to avoid. In other words, by coming with some expectations about proper conduct, it may challenge local authorities and agreed-upon ways to do systematic reviews. In contrast to clinical practice, the implementation of reporting guidelines provides some level of enforcement that limits the individual flexibility to divert from the standard in practice (see Timmermans and Berg, 2003). To preserve local autonomy, biomedical subdisciplines have developed extensions and forks that make PRISMA more suitable to particular research cultures. Currently, there are twelve extensions that tweak the guideline toward practices such as equity research, network meta-analyses, or usages of individual patient data (Page and Moher, 2017). In addition, other scientific disciplines have developed different reporting guidelines altogether. For example, the EQUATOR network currently lists nearly five-hundred different reporting standards (www.equator-network.org) This shows that standardizing the reporting of research may be all but a one-size-fits-all approach.

## Conclusion

This study investigated how the PRISMA reporting guideline attempts to transform transparency into a new quality goal for clinical biomedicine. Besides providing an in-depth analysis of the guideline and the development of its various unique characteristics, this study showed how transparency as a scientific value or quality goal can become manifest in the form of a codified standard and what gets lost on that way. With respect to the research questions, three aspects shall be highlighted.

First, in an attempt to standardize scientific practices, PRISMA perpetuates the homogeneity of systematic reviews and thereby shapes the boundaries of clinical medicine. It redefines what counts as legitimate evidence for evidence-based medical practice by making non-complying systematic reviews unpublishable and weeding out the landscape of medical genres. Being a standard that is implemented where science is usually evaluated, PRISMA makes visible how ex post and ex ante evaluation are inseparably connected. It is not only observable how researchers adapt to this form of evaluation and change their behavior, rather the advocates of reporting guidelines and the developers of PRISMA explicitly intended this development.

Second, PRISMA and transparency as a value forged strong bonds in order to create a convincing and powerful narrative. While transparency as a universal value has acquired substantial normative momentum among various biomedical disciplines and practices, PRISMA and other reporting guidelines had to disentangle the research process in order to become applicable. As such, especially the reporting or writing of systematic reviews was turned into a well-defined practice that can be evaluated and managed independently of the overall research process. This set the realms and boundaries in which transparency can be evaluated and thereby ultimately turned this value into a meaningful quality goal at all.

Third, the actual formulation of the guideline and its items does violate the boundaries of transparency as traditionally defined. In fact, transparency as a quality goal gained momentum because it promises to avoid conflicts over values by not solving them. Rather, transparency just relocates such evaluations. At the same time, the PRISMA guideline as a practical manifestation of transparency indeed claims to support evaluation and closure. It offers authors, editors and reviewers alike to serve as a proxy for a particular quality that can be achieved and monitored. In doing so, it must define what authors have to report transparently and thereby gives prominence to value judgements other than transparency itself.

## Data Availability Statement

The data that support the findings of this study are available on request from the corresponding author, AS. Although interview participants agreed with the sharing and reuse of the data, full publication of data beyond the quotes provided in this text is not intended due to economic and organizational constraints.

## Author Contributions

Conceptualization and writing was done by AS.

## Funding

This work was supported by the German Ministry of Education and Research (BMBF) under Grant FKZ: 01PU17017. Additional support was provided by the German Kompetenzzentrum Bibliometrie (01PQ17001).

## Conflict of Interest

The author declares that the research was conducted in the absence of any commercial or financial relationships that could be construed as a potential conflict of interest.

## Publisher's Note

All claims expressed in this article are solely those of the authors and do not necessarily represent those of their affiliated organizations, or those of the publisher, the editors and the reviewers. Any product that may be evaluated in this article, or claim that may be made by its manufacturer, is not guaranteed or endorsed by the publisher.

## Footnotes

^1^The multipublished versions differ only marginally. In the following, PRISMA is referred to in singular because every document represents the same guideline. Exact references are made to the version published in the British Medical Journal (BMJ).
